# Random Forest Classifiers to predict psychotic symptoms in Alzheimer’s disease

**DOI:** 10.1002/alz.092242

**Published:** 2025-01-03

**Authors:** Sara Scarfo, Yashar Moshfeghi, William J McGeown

**Affiliations:** ^1^ University of Strathclyde, Glasgow United Kingdom

## Abstract

**Background:**

Psychotic symptoms (delusions and/or hallucinations) are among the most common and impactful neuropsychiatric symptoms that occur within Alzheimer’s disease (AD): present in around half of AD patients, they are associated with increased distress for the individual and their families, poorer disease outcome, and greater risk of hospitalization and death. Using Random Forest analyses, aim of this study is to provide clarity on the value of neuroanatomical, neuropsychological, and neuropsychiatric features when predicting the presence of psychotic symptoms in AD.

**Method:**

Data used in preparation for this study was obtained from the Alzheimer’s Disease Neuroimaging Initiative database (adni.loni.usc.edu). Based on the scores of the Neuropsychiatric Inventory, participants were selected with the criterion of presenting with psychotic symptoms (either delusions, or hallucinations, or both). Controls were subsequently matched, who did not present with any psychotic symptom, and did not differ from the psychosis group for disease stage, age, gender, education, genetic profile, and overall cognitive and neuropsychiatric status. The predictors were derived from: Neuropsychiatric Inventory’s scores for the other neuropsychiatric symptoms; Alzheimer’s Disease Assessment Scale‐Cognitive Subscale, to select different cognitive functions; brain metrics derived from the FreeSurfer software (available via ADNI), as measures of cortical and subcortical volumes, cortical thickness, and surface area.

**Result:**

The Random Forest Classifiers were employed to generate feature importance ranking. While further analyses are currently underway, preliminary results suggest that it is possible to understand how the different variables interact to predict the presence of psychotic symptoms, and which of those variables hold greater importance. Among neuropsychiatric symptoms, apathy has emerged; short‐term and long‐term memory and orientation resulted as best predictors among the cognitive functions. Lastly, the anterior and posterior cingulate cortex and insula (bilaterally), and right frontal areas were identified as the most important brain areas. Overall, the brain region metrics appear to have better discriminatory ability compared to neuropsychological and neuropsychiatric data.

**Conclusion:**

The results of this study indicate (albeit preliminarily) that a machine learning technique such as the Random Forest Classifier can be used to advance our understanding of the complex interaction of different predictors in the manifestation of psychotic symptoms in AD.

